# Predictive Value of Fever and Palmar Pallor for *P. falciparum* Parasitaemia in Children from an Endemic Area

**DOI:** 10.1371/journal.pone.0036678

**Published:** 2012-05-04

**Authors:** Christof David Vinnemeier, Norbert Georg Schwarz, Nimako Sarpong, Wibke Loag, Samuel Acquah, Bernard Nkrumah, Frank Huenger, Yaw Adu-Sarkodie, Jürgen May

**Affiliations:** 1 Infectious Disease Epidemiology, Bernhard-Nocht-Institute for Tropical Medicine, Hamburg, Germany; 2 Kumasi Centre for Collaborative Research in Tropical Medicine, Kumasi, Ghana; 3 School of Medical Sciences, Kwame Nkrumah University of Science and Technology, Kumasi, Ghana; 4 Institute for Transfusion Medicine, Laboratory Medicine and Medical Microbiology, Dortmund, Germany; Menzies School of Health Research, Australia

## Abstract

**Introduction:**

Although the incidence of *Plasmodium falciparum* malaria in some parts of sub-Saharan Africa is reported to decline and other conditions, causing similar symptoms as clinical malaria are gaining in relevance, presumptive anti-malarial treatment is still common. This study traced for age-dependent signs and symptoms predictive for *P. falciparum* parasitaemia.

**Methods:**

In total, 5447 visits of 3641 patients between 2–60 months of age who attended an outpatient department (OPD) of a rural hospital in the Ashanti Region, Ghana, were analysed. All Children were examined by a paediatrician and a full blood count and thick smear were done. A Classification and Regression Tree (CART) model was used to generate a clinical decision tree to predict malarial parasitaemia a7nd predictive values of all symptoms were calculated.

**Results:**

Malarial parasitaemia was detected in children between 2–12 months and between 12–60 months of age with a prevalence of 13.8% and 30.6%, respectively. The CART-model revealed age-dependent differences in the ability of the variables to predict parasitaemia. While *palmar pallor* was the most important symptom in children between 2–12 months, a *report of fever* and an *elevated body temperature* of ≥37.5°C gained in relevance in children between 12–60 months. The variable *palmar pallor* was significantly (p<0.001) associated with lower haemoglobin levels in children of all ages. Compared to the Integrated Management of Childhood Illness (IMCI) algorithm the CART-model had much lower sensitivities, but higher specificities and positive predictive values for a malarial parasitaemia.

**Conclusions:**

Use of age-derived algorithms increases the specificity of the prediction for *P. falciparum* parasitaemia. The predictive value of *palmar pallor* should be underlined in health worker training. Due to a lack of sensitivity neither the best algorithm nor *palmar pallor* as a single sign are eligible for decision-making and cannot replace presumptive treatment or laboratory diagnosis.

## Introduction

According to the latest figures of the World Health Organization (WHO) there were 225 million cases of malaria estimated for the year 2009, responsible for 781000 deaths [1]. Although decreasing numbers of malaria cases and mortality are reported in all WHO Regions, Africa remains the continent with the largest proportion of deaths caused by the disease [1]–[4]. In 2009, ninety-one percent of malaria fatalities worldwide occurred in Africa, 85% of these deaths were in children under 5 years of age [5]. The WHO implemented the Integrated Management of Childhood Illness (IMCI) Programme in 1997 as a systematic approach to children's health in response to increasing numbers of deaths in children under five years of age [6].

The IMCI-system should enable health workers in rural areas to identify severely ill children using simple evidence based clinical algorithms [7]. Since the beginning of the programme improvements of health care quality with an effect on child health have been reported [8]–[10]. During the 90's and the early first decade of 2000 several attempts have been undertaken to establish clinical algorithms that enable health workers in poor settings to diagnose malaria based on clinical features in children [11]–[16]. The IMCI-algorithm bases on a few symptoms and parameters with high sensitivities but low specificities for the classification of malaria. Consequently, over-treatment and drug wastage were frequent points of criticism [2], [17], [18].

Within the last 14 years after implementation of IMCI in 44 of 46 African countries, some of the general conditions in these countries have changed [19]. Health worker performance improved, health care seeking behaviour of parents was enhanced and distribution of anti-malarials, antipyretics and insecticide treated bed-nets (ITNs) was extended [1], [2], [20]. On the other hand, the proportion of other diseases presented by children in health facilities with overlapping symptoms to malaria has been increasing [1], [2], [21].

The aim of the present study is to generate an age-derived clinical algorithm with simple signs and symptoms for the diagnosis of *Plasmodium falciparum* parasitaemia.

An algorithm such as this could help to detect focus groups of children for presumptive treatment if resources are scarce. For better adoption to the clinical decision-making, we used a Classification and Regression Tree model (CART).

## Methods

### Study setting

This study was conducted in Agogo, Ghana, which is located in the Agogo District, 80 kilometres east of the regional capital Kumasi. The population in the Agogo District and the neighboured Ashanti Akim North District is estimated with 140.000. The climate is tropical, and the entire Ashanti region is holoendemic for malaria. Transmission of parasites is stable with an estimated entomological inoculation rate (EIR) of >400 per year [22]. The predominant malaria parasite is *P. falciparum* [23]. The Agogo Presbyterian Hospital (APH) is a 250-bed facility, divided in five departments and employs 19 doctors.

### Data collection

Children aged up to 5 years who visited the Outpatient Department for any illness in the period between May 2007 and July 2009 were included in the study. Body temperature, weight and height were measured and documented in a Case Report Form (CRF) with predefined clinical criteria by trained study nurses. Afterwards a pediatrician interviewed the caretaker of the child and carried out a physical examination. Three nurses and two doctors were working in the OPD at the same time. All clinical symptoms were assessed before health personnel had access to laboratory data.

A *report of fever* was defined as any history of intermittent or continuous fever or ‘feeling hot’ within the last five days. An *elevated body temperature* was defined as a temperature of ≥37.5°C or above. An EDTA-blood sample was collected for full blood count and Giemsa-stained thick and thin blood films. The outcome of the CART models was *P. falciparum* parasitaemia of any density.

### Data Management and processing for database

A patient was defined as an individual visiting the OPD. Every individual visiting the OPD without a previous visit within 14 days was included as a “new patient”.

CRF and laboratory results were double entered within 48 hours into a database (4^th^ Dimension, 4D SAS, France). Inconsistencies were checked by a data manager. Statistical analyses were carried out using STATA IC/10.0 statistical and data analysis software (Stata Cooperation, California).

**Figure 1:**
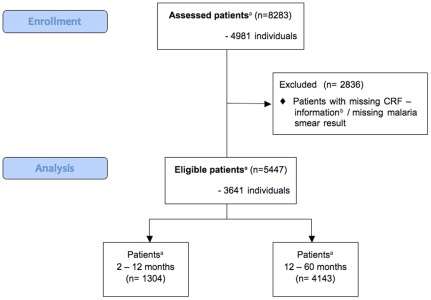
Enrollment and exclusion of patients for analysis. ^a^ A patient is defined as an individual visiting the OPD. ^b^ Case report forms must have information for each variable in Table 2 available.

Of 8283 visits of 4981 individuals during the study period, only those were included in the calculations, which had a malaria smear result available (Table 1, Figure 1). Cases that had missing values for one of the variables in Table 2 were also not included in further analyses. After exclusion of cases with incomplete information, 5447 visits of 3641 individuals were left.

**Table 1:** Characteristics of 5447^b^ patient visits in the study.

		Total	No Parasites detected^a^	Parasites detected^a^
		n = 5447^b^	n = 3998^b^	n = 1449^b^
Sex	Male (%)	2882	2112 (73.3)	770 (26.7)
	Female (%)	2565	1886 (73.5)	679 (26.5)
Age, months	2–12 (%)	1304	1124 (86.2)	180 (13.8)
	12–60 (%)	4143	2874 (69.4)	1269 (30.6)
Residence	Agogo (%)	2526	1768 (70.0)	758 (30.0)
	Outside Agogo (%)	2921	2230 (76.3)	691 (23.7)

a Presence of *P. falciparum* parasites was analyzed by thick blood smear or thin blood film.

b 5447 visits of 3641 individuals were included in the study.

**Table 2:** Signs and symptoms and their association with *P. falciparum* parasitaemia in children.

Signs and Symptoms	Patient visits		No parasites detected		Parasites detected		Odds Ratio	95% – CI^e^	p
	N = 5447		N = 3998		N = 1449				
	n	(%)	n	(%)	n	(%)			
**Signs or symptoms assessed by nurse or doctor**									
Running Nose	3074	(56.4)	2466	(61.7)	608	(42.0)	0.44	0.39–0.50	<0.001
Elevated body temperature	2025	(37.2)	1143	(28.6)	882	(60.9)	1.97	1.86–2.09	<0.001
Blocked nose	762	(14.0)	631	(15.8)	131	(9.0)	0.53	0.43–0.64	<0.001
Skin rash	621	(11.4)	530	(13.3)	91	(6.3)	0.43	0.34–4.52	<0.001
Palmar pallor	477	(8.8)	218	(5.5)	259	(17.9)	2.80	2.38–3.30	<0.001
Skin abnormalities	344	(6.3)	268	(6.7)	76	(5.2)	0.77	0.59–1.00	0.051
Other skin problem	179	(3.3)	122	(3.1)	57	(3.9)	1.30	0.94–1.79	0.107
Malnourished condition	136	(2.5)	110	(2.8)	26	(1.8)	0.64	0.41–0.99	0.047
Prostration	115	(2.1)	61	(1.5)	54	(3.7)	2.49	1,72–3,62	<0.001
Fast breathing	88	(1.6)	72	(1.8)	16	(1.1)	0.60	0.35–1.05	0.074
Jaundice	82	(1.5)	35	(0.9)	47	(3.2)	3.79	2.44–5.90	<0.001
Breathing difficulties	60	(1.1)	50	(1.3)	10	(0.7)	0.54	0.27–1.08	0.084
Chest indrawing	59	(1.1)	46	(1.2)	13	(0.9)	0.77	0.41–1.44	0.426
Respiratory distress	58	(1.1)	40	(1.0)	18	(1.2)	1.24	0.71–2.17	0.443
Skin depigmentation	51	(0.9)	45	(1.1)	6	(0.4)	0.36	0.15–0.85	0.021
Deep Breathing	33	(0.6)	24	(0.6)	9	(0.6)	1.03	0.48–2.23	0.930
Pinch abdomen present	21	(0.4)	20	(0.5)	1	(0.1)	0.13	0.01–1.02	0.053
Cyanosis	1	(0.01)	0	(0)	1	(0.1)	0.91	0.09–8.79	0.938
**Signs or symptoms taken from history by child, parent or legal guardian**									
Report of fever	3859	(70.8)	2647	(66.2)	1212	(83.6)	2.61	2.23–3.04	<0.001
Nutrition changed to poor feeding	3659	(67.2)	2617	(65.5)	1042	(71.9)	1.32	1.16–1.50	<0.001
Cough	3081	(56.6)	2500	(62.5)	581	(40.1)	0.40	0.35–0.45	<0.001
Vomiting	1870	(34.3)	1275	(31.9)	595	(41.1)	1.48	1.31–1.68	<0.001
Diarrhoea	1636	(30)	1264	(31.6)	372	(25.7)	0.74	0.65–0.85	<0.001
Drinking thirsty	425	(7.8)	284	(7.1)	141	(9.7)	1.40	1.14–1.74	0.001
Earpain	172	(3.2)	135	(3.4)	37	(2.6)	0.74	0.51–1.08	0.126
Convulsions	20	(0.4)	12	(0.3)	8	(0.6)	1.84	0.75–4.52	0.180
**Generated signs or symptoms**									
No malnourishment^a^	5311	(97.5)	3888	(97.2)	1423	(98.2)	1.55	1.01–2.38	0.047
No skin symptoms^b^	4687	(86.1)	3374	(84.4)	1313	(90.6)	1.79	1.47–2.17	<0.001
No gastrointestinal symptoms^c^	2682	(49.2)	2019	(50.5)	663	(45.8)	0.82	0.73–0.93	0.002
No respiratory symptoms^d^	1639	(30.1)	1002	(25.1)	637	(44.0)	2.34	2.06–2.66	<0.001

a To be positive for this (inverse) variable patients must not present *malnourished condition*.

b To be positive for this (inverse) variable patients must not present *skin abnormalities*, *skin rash*, *skin depigmentation* and *other skin problem*.

c To be positive for this (inverse) variable patients must not present *vomiting* and *diarrhoea*.

d To be positive for this (inverse) variable patients must not present *respiratory distress, breathing difficulties, fast breathing, deep breathing, chest indrawing, running nose*, blocked nose and *cough*.

e CI: 95% Confidence interval.

### Data analysis

Each variable from Table 2 was put in a bivariate regression analysis to compute Odds Ratios between symptoms and parasitaemia. The resulting Odds Ratios were used to decide whether the variables are handled as potentially predictive. Those variables providing both, an Odds Ratio ≤0.83 or ≥1.20 and an occurrence in at least 1% of cases with present parasitaemia were put in a forward and a backward stepwise logistic regression. For variables with an Odds Ratio ≤0.83 bunched inverse variables were generated (e.g. no respiratory symptoms).

For stepwise estimation a p-value of <0.05 was set as a statistical cut-off. Variables that were significantly associated with parasitaemia were then entered in the CART – analysis for the generation of an algorithm, which could be suitable for clinical routine.

Beginning with a parent node containing all cases of a specific age group, CART is continuously trying to maximize the purity, i.e. to segregate children who are affected by parasitaemia from those children who are not affected. Therefore, the model generates a decision tree by splitting the cases of the parent node at an optimal split point where the arising child nodes provide greater purity (better segregation of affected and non-affected children). For detection of the optimal split point the entered binary prediction variables were used.

Every child node in turn can become a parent node, as CART continues the splitting process until statistical analysis indicates that another child node would not be of greater purity [24], [25]. The resulting tree is structured as a sequence of yes/no questions. For calculation of cut-points a p-value of <0.05 and a minimum size of the subgroup of 10 cases were set. CART-analyses were separately carried out for two different age groups (2–12 months and 12–60 months).

Sensitivity, specificity, negative (NPV) and positive (PPV) predictive values were calculated for each CART-model and compared to the IMCI-algorithm. Haemoglobin (Hb)-values of children with *palmar pallor* were compared to those without. For testing the null hypothesis on this variable an unpaired two-sample t-test was performed, assuming a Student's t distribution. To discover potiential over-representation of *palmar pallor* due to the effect of multipe visits of single individuals a McNemar test was used. Additionally, an alternative CART model was created for both age groups containing only the first visit of every individual.

### Methodology of laboratory examinations

For analysis of the capillary blood samples, a Sysmex® KX-21N Haematological Analyzer (Sysmex Corporation, Kobe, Japan) was used. The machine was tested and calibrated on a daily basis. A three level control (Eightcheck-3WP controls (Low, Normal and High)) were run each morning to ensure the function of the machine.

Thick and thin blood smears were prepared for each child enrolled in the study, stained with 10% Giemsa working solution and examined by using immersion oil microscopy with 100× magnification for detection, species identification and quantification of malaria parasites. The number of *P. falciparum* parasites per 200 white blood cells (thick smear) or in case of very high parasitaemia per 1000 erythrocytes (thin film) was assessed and densities were recorded as the number of parasites/µl of blood.

As predictive signs and symptoms for *P. falciparum* parasitaemia were traced, a child was considered to be parasitaemic at any density of parasites, i.e. there was no parasite density threshold set. If there was no blood count available, an average of 8000 leucocytes per µl of blood was assumed [26].

### Consent and Ethical Approval

The study was conducted in accordance with the ethical principles of the Declaration of Helsinki, and consistent with Good Clinical Practice (GCP). Ethical approval for the study was obtained from the Ethics committees of the School of Medical Science, Kwame Nkrumah University of Science and Technology (KNUST), Kumasi.

Mothers or guardians of children were informed about the aim of the study in presence of a witness and their understanding was assessed by a set of standard questions. Informed consent was sought from mothers who granted it by signature or thumbprint.

## Results

### Describing the population

We included 5447 patients under 5 years of age (3641 individuals) in the analyses, 52.9% were male. About 23.9% of all children were between 2–12 months old and 76.1% of children between 12–60 months. The prevalence of *P. falciparum* parasitaemia increased with age from 13.8% to 30.6% (Table 1).

### Predictive value of clinical symptoms

Table 2 shows the clinical symptoms for prediction of parasitaemia in a bivariate regression analysis. All variables with both characteristics, an Odds Ratio ≤0.83 or ≥1.20 and an occurrence in at least 1% of cases with present parasitaemia, were entered in the forward and backward stepwise estimation (not displayed). Both parameter reduction methods revealed the following variables to be able to predict a parasitaemia: *Palmar pallor* with an OR of 3.06 (95%-CI: 2.49–3.78; p<0.001), *elevated body temperature* with an OR of 2.82 (95%-CI: 2.47–3.23; p<0.001), *report of fever* with an OR of 4.62 (95%-CI: 3.39–6.30; p<0.001), *other skin problem* with an OR of 2.25 (95%-CI: 1.47–3.47; p<0.001), *vomiting* with an OR of 1.33 (95%-CI: 1.16–1.52; p<0.001), *no respiratory symptoms* with an OR of 2.45 (95%-CI: 2.14–2.81; p<0.001), *no skin symptoms* with an OR of 1.97 (95%-CI: 1.51–2.56; p<0.001) and *no malnourishment* with an OR of 1.71 (95%-CI: 1.08–2.76; p<0.025). These variables were then included in the CART-analyses.

### CART-analysis

**Figure 2:**
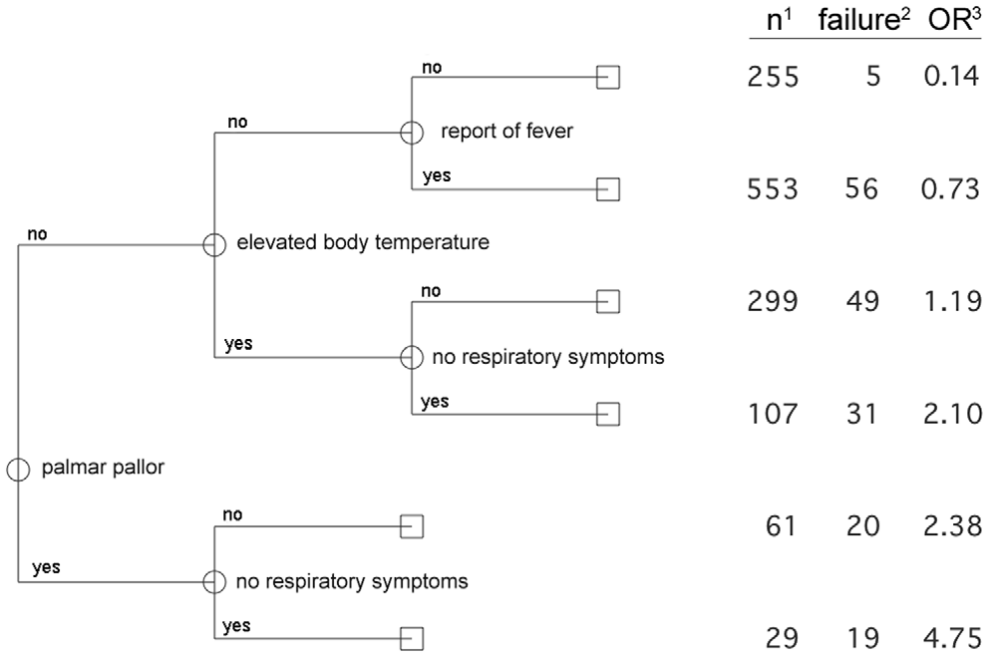
CART – model for children between 2 and 12 months of age (N = 1304). ^1^ Number of patients with the respective combination of variables given by the branches of the decision tree. ^2^ Number of patients positive for *P. falciparum* parasitaemia. ^3^ Odds Ratio for *P. falciparum* parasitaemia with the combination of variables in comparison to all other combinations.

The CART-analysis was performed separately for the two age groups. In children between 2–12 months of age, the first decision was made by whether the patient was diagnosed with *palmar pallor* or not (Figure 2). In 19 children out of 29 who presented with *palmar pallor* but without respiratory symptoms (*no respiratory symptoms* = 1) a *P. falciparum* parasitaemia was detected (OR = 4.75). To the contrary, patients without *palmar pallor*, without an *elevated body temperature* (≥37.5°C) on admission and without any *report of fever* in the last five days had an OR of 0.14 for parasitaemia. For those children, who had a positive *report of fever*, the likelihood of having parasitaemia was slightly higher with an OR of 0.73.

**Figure 3:**
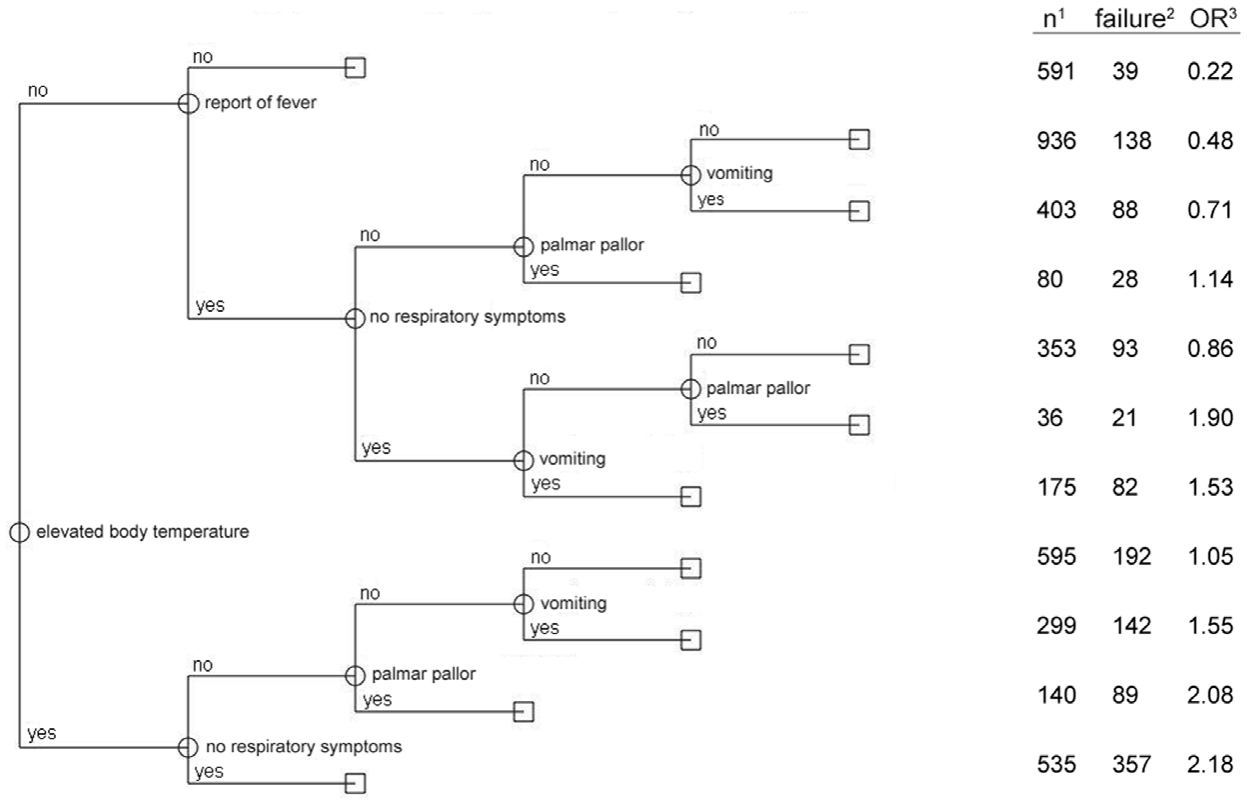
CART – model for children between 12 and 60 months of age (N = 4143). ^1^ Number of patients with the respective combination of variables given by the branches of the decision tree. ^2^ Number of patients positive for *P. falciparum* parasitaemia. ^3^ Odds Ratio for *P. falciparum* parasitaemia with the combination of variables in comparison to all other combinations.

Compared to the CART-model for children between 12–60 months several differences were obvious (Figure 3). While *palmar pallor* was strongly predictive for parasitaemia in children between 2–12 months of age, it generated only small differences of the OR in older children where *elevated body temperature* was the first decisive variable. The lowest likelihood for a parasitaemia was found in children without *elevated body temperature* and without *report of fever* (OR of 0.22 for malarial parasitaemia). On the other hand an *elevated body temperature* and absence of respiratory symptoms resulted in the highest risk for parasitaemia (OR 2.18).

**Figure 4:**
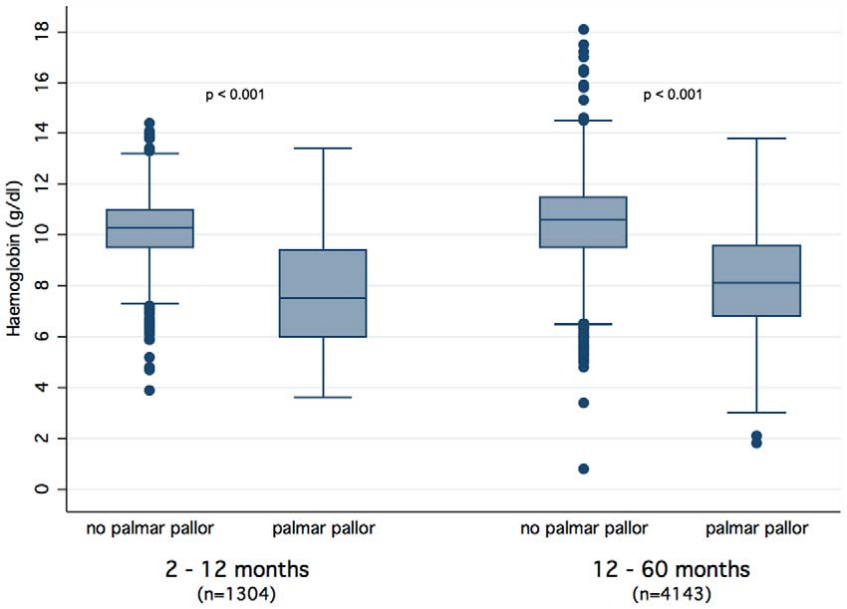
Distribution of haemoglobin-values in patients of different ages with and without palmar pallor. p-value was calculated assuming a Student's t distribution.

As expected, Hb levels were significantly lower for those children who were diagnosed with *palmar pallor* (Figure 4). Children aged between 2 and 12 months had a mean Hb level of 7.7 g/dl if *palmar pallor* was present and 10.2 g/dl if not (p<0.001). Children aged between 12 and 60 months with *palmar pallor* and those without had a mean Hb of 8.1 g/dl and 10.4 g/dl, respectively (p<0.001). The McNemar-test showed no heterogeneity (p = 0.343) for the symptom *palmar pallor* between patients who had only one visit to the OPD and those patients who came for more than one time (Table S1). An alternative CART-model that included only the first visit of every individual also showed no deviance from the original models (Figure S1 and S2).

In Table 3 data on the sensitivity and the specificity for the prediction of *P. falciparum* parasitaemia are shown for the five variables that remained in the CART analyses. In both age groups the proportion of children with a *report of fever* is larger than the proportion of children who are actually presenting with an *elevated body temperature* on admission. In both age-derived models, *palmar pallor* has the highest specificity of all variables for predicting a malarial parasitaemia.

**Table 3:** Sensitivity and specificity of symptoms for prediction of *P. falciparum* parasitaemia in different age groups.

	Patients	2–12 months	12–60 months
ROF^a^	N (%)	1029 (78.9)	3539 (85.4)
	Sens./Spec.	96.0/23.8	96.8/19.6
EBT^b^	N (%)	465 (35)	1569 (37.9)
	Sens./Spec.	56.7/68.5	61.5/72.5
Palmar Pallor	N (%)	90 (6.9)	387 (9.3)
	Sens./Spec.	21.7/95.5	17.3/94.2
Vomiting	N (%)	549 (42.1)	1321 (31.9)
	Sens./Spec.	40.0/57.6	41.2/72.2
NRS^c^	N (%)	340 (26.1)	1299 (31.6)
	Sens./Spec.	38.9/76	44.7/74.5

Note: Percentage refers to the total number of patients within each age group.

a ROF = *report of fever*;

b EBT = *elevated body temperature*;

c NRS = *no respiratory symptoms*.

### Classification and comparison of the CART-model and the IMCI-algorithm

For calculation of sensitivity, specificity, positive- and negative predictive value, we created a logistic regression model containing all variables used in the corresponding model (Table 4). Regarding children between 2–12 months, the algorithm provided in the IMCI system gains a much higher sensitivity of 97.2% than the CART-model (6.7%), but remains unspecific in the prediction of parasitaemia with a specificity of 22.2% versus 99.6% in the CART-model. For the older children these relations were decreasing, as the IMCI-algorithm provided a sensitivity of 55.6% and a specificity of 73.4% vs. 37.7% and 91.4% for the CART-model, respectively.

**Table 4:** Classification and comparison of CART model and IMCI algorithm.

Age (months)	Model	Sensitivity (%)	Specificity (%)	PPV^c^ (%)	NPV^d^ (%)
2–12	IMCI-algorithm^a^	97.2	22.2	16.7	98.0
(n = 1304)	CART-model^b^	6.7	99.6	75.0	87.0
12–60	IMCI-algorithm^a^	55.6	73.4	48.0	78.9
(n = 4143)	CART-model^b^	37.7	91.4	65.8	76.9

a IMCI-algorithm for identification of children with malaria in high-risk areas: Fever by history of fever or feeling hot/elevated body temperature of ≥37.5°C on admission and/or some palmar pallor.

b CART-model: For calculation only those variables were used, which were included in the CART-analysis for the certain age group.

c PPV = Positive predictive value.

d NPV = Negative predictive value.

Overall, the CART-model results in much higher specificities and higher positive predictive values for prediction of malarial parasitaemia in comparison to the IMCI-model, which provides higher sensitivities and negative predictive values.

## Discussion

We aimed to generate a simple clinical algorithm to predict malarial parasitaemia in sick children attending a health facility by means of a Classification and Regression Tree (CART) model. The hierarchical CART-based decision algorithm increased the prediction specificity for malarial parasitaemia in comparison to a simple combination of single parameters. However, despite of a good specificity of the best model, a significant proportion of children with malaria would have been overseen with this clinical algorithm due to a lack of appropriate sensitivity. The use of clinical algorithms cannot replace laboratory diagnosis or RDT's for treatment decisions. In case of unavailability of these tests, presumptive treatment of children with suspected malaria is necessary.

A clinical algorithm, given the predictive values are appropriate, would facilitate the decision for presumptive treatment of certain focus groups. Apart from earlier studies, we observed all children between 2 and 60 months of age attending an OPD during the study period. To gain more knowledge about the relevance of the symptom fever for the prediction of parasitaemia, we decided not to use ‘fever’ or a ‘history of fever’ as an inclusion criterion. Instead, all children visiting the OPD were included. Accordingly, the primary outcome was defined as ‘*Plasmodium falciparum* parasitaemia’ instead of ‘clinical malaria’, which is commonly defined as presence of parasites and fever or a history of fever [11], [13], [15].

Compared to previous investigations, we recorded a relatively low prevalence of malaria parasitaemia, which may have been due to the fact, that we enrolled all children who presented to the OPD for any illness during the study period, disregarding any prerequisites [27]–[29]. Children (12–60 months) were more affected by parasitaemia than infants (2–12 months), which is in line with earlier findings.

In contrast to other studies a CART model was used, a method discussed controversially. Nevertheless, CART is – compared to multiple logistic regression – expected to be more suitable for data where interactions occur [30].

Beyond five symptoms that were used by the CART-model, the symptoms *palmar pallor, elevated body temperature* on admission and *report of fever* revealed age-dependent differences in the ability to predict malarial parasitaemia.

Pallor caused by anaemia is a common sign in SSA, in our study it was noticed in 8.8% of all cases [31]–[33]. The impact of *palmar pallor* through all CART algorithms revealed the importance of the symptom as predictor of *P. falciparum* infection. In the model for the youngest children (Figure 2) *palmar pallor* was even selected as the first decisive variable. Investigation of full blood counts showed, that *palmar pallor* could predict parasitaemia even in children from this population with a high prevalence of anaemia. Indeed, children with *palmar pallor* had significantly lower Hb-levels than those who were not diagnosed as being pale on their palms (Figure 4). Previous studies showed diverse results on the ability of health workers to recognize pallor [32], [34], [35]. Health workers in high malaria transmission areas had less difficulties [36].

We considered two different fever definitions. Although it provides higher sensitivity, a *report of fever* was of lower priority in all CART-models, than an *elevated body temperature*. In children below the age of 12 months, fever did not count for the branch of our CART model with the highest predictivity. Even though studies demonstrated that mothers are able to identify an elevated body temperature in their children with sensitivites up to 76%, a *report of fever* given by the caretaker remains relatively unspecific [37]. Our data showed specificities below 24% for the variable *report of feve*r predicting *P. falciparum* parasitaemia in both age groups. Most of the febrile episodes reported could not be confirmed with thermometers on admission, probably due to the use of antipyretics. This finding was also observed in other studies [13], [38].

Application of the CART algorithm showed that the predictive value of fever was increasing with the age of the children. In contrast, *palmar pallor* had a much higher impact in infants. However, these symptoms were applied in the context of an algorithm and as in previous studies we can confirm that higher developed algorithms are able to provide much higher specificities, when compared to the IMCI-algorithm, but lack in sensitivity. For children between 2 and 12 months IMCI gives a sensitivity of 97.2% and a specificity of 22.2%, CART provides 6.7% and 99.6%, respectively. Vital for the decision of the health worker and for the health of the patient are the predictive values, which are dependent on the prevalence of the outcome. With decreasing prevalences of malaria the negative predictive value will increase and the positive predictive value will decrease given the same sensitivity and specificity of the algorithm. Comparison of our approach to IMCI may be aggravated as our outcome was *P. falciparum* parasitaemia and IMCI mainly targets clinical malaria. The algorithm itself is not eligible to make treatment decisions without laboratory support or even to withheld anti-malarials to children with suspected malaria.

During the last two years there is an ongoing debate, whether to move away from presumptive treatment of febrile children for malaria to laboratory-confirmed treatment. Proponents mainly refer to the substantial decline of malaria transmission and the availability of reliable rapid diagnostic tests (RDT's) [2], [39]–[41] while others raised the concern that health system capacity is not yet sufficient to implement such a policy change [42].

Health workers in primary health care indeed play a key role for correct assessment and treatment of children with malaria. They are frequently forced to decide about treatment under great pressure, because often children are presented to them only once, due to access barriers to hospitals such as long travel distances, costs or lack of health insurance coverage [43]. As our results show, it would be appropriate to sensitize health workers for the sign of *palmar pallor* as a symptom possibly predicting malarial parasitaemia, especially in infants. However, the symptom is not eligible to be applied for making a treatment decision. Instead of this, continuous reviews of patients could lead to more conservative treatment strategies. It is further necessary to keep on track with distribution of equipment for malaria diagnostics.

### Conclusions

The value of the symptoms fever and palmar pallor to predict *P. falciparum* parasitaemia is age-dependent. Palmar pallor is easy to recognize and might be helpful for health workers as an indicator not only for anaemia but also for malarial parasitaemia whereas this clinical sign cannot replace thorough laboratory diagnostics.

## Supporting Information

Figure S1 CART – model for the first visit* of children between 2 and 12 months of age (N = 1031). ^*^ CART-model was calculated only for the first visit of each individual. Subsequent visits of individuals were excluded from the analysis. ^1^ Number of patients with the respective combination of variables given by the branches of the decision tree. ^2^ Number of patients positive for *P. falciparum* parasitaemia. ^3^ Odds Ratio for *P. falciparum* parasitaemia with the combination of variables in comparison to all other combinations.
(TIF)

Figure S2 CART – model for the first visit* of children between 12 and 60 months of age (N = 2610). ^*^ CART-model was calculated only for the first visit of each individual. Subsequent visits of individuals were excluded from the analysis. ^1^ Number of patients with the respective combination of variables given by the branches of the decision tree. ^2^ Number of patients positive for *P. falciparum* parasitaemia. ^3^ Odds Ratio for *P. falciparum* parasitaemia with the combination of variables in comparison to all other combinations.
(TIF)

Table S1 Re-occurrence of *palmar pallor* in 1125 patients who had multiple visits (n = 2931) to the Outpatient department. McNemar-test: chi^2^: 0.90; degrees of freedom:1; Prob>chi^2^: 0.343.
(DOC)
