# Supplementary figures and images for: Predictive Value of Fever and Palmar Pallor for P. falciparum Parasitaemia in Children from an Endemic Area
Source: PLoS One. 2012 May 4;7(5):e36678. doi: 10.1371/journal.pone.0036678 (PMC3344934; doi:10.1371/journal.pone.0036678)

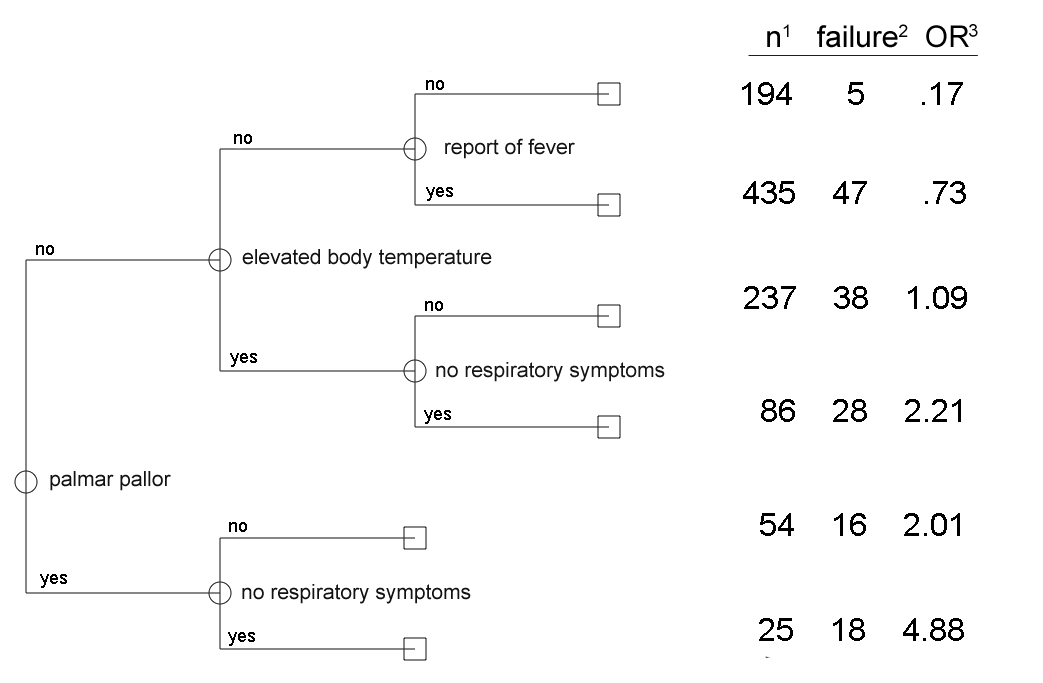

Supplement: Figure S1 — CART – model for the first visit* of children between 2 and 12 months of age (N = 1031). * CART-model was calculated only for the first visit of each individual. Subsequent visits of individuals were excluded from the analysis. 1 Number of patients with the respective combination of variables given by the branches of the decision tree. 2 Number of patients positive for P. falciparum parasitaemia. 3 Odds Ratio for P. falciparum parasitaemia with the combination of variables in comparison to all other combinations. (TIF) [file pone.0036678.s001.tif]

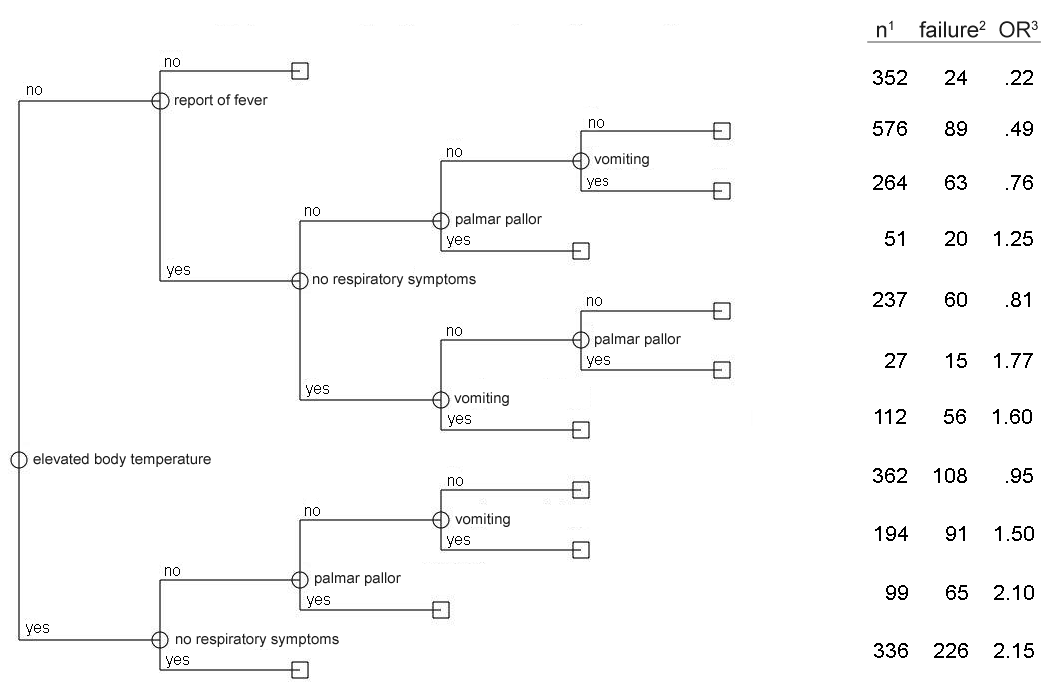

Supplement: Figure S2 — CART – model for the first visit* of children between 12 and 60 months of age (N = 2610). * CART-model was calculated only for the first visit of each individual. Subsequent visits of individuals were excluded from the analysis. 1 Number of patients with the respective combination of variables given by the branches of the decision tree. 2 Number of patients positive for P. falciparum parasitaemia. 3 Odds Ratio for P. falciparum parasitaemia with the combination of variables in comparison to all other combinations. (TIF) [file pone.0036678.s002.tif]
