# Supplementary material for: Predictive Value of Fever and Palmar Pallor for P. falciparum Parasitaemia in Children from an Endemic Area
Source: PLoS One. 2012 May 4;7(5):e36678. doi: 10.1371/journal.pone.0036678 (PMC3344934; doi:10.1371/journal.pone.0036678)
Supplement: Table S1 — Re-occurrence of palmar pallor in 1125 patients who had multiple visits (n = 2931) to the Outpatient department. McNemar-test: chi2: 0.90; degrees of freedom:1; Prob>chi2: 0.343. (DOC) [file pone.0036678.s003.doc]

Supportive information

**Table S1.** Re-occurrence of *palmar pallor* in 1125 patients who had multiple visits

(n=2931) to the Outpatient department

| Palmar Pallor on first visit | Pallor on subsequent visit | | Total |
| --- | --- | --- | --- |
|  | No | Yes |  |
| No | 2662 (95.8%) | 117 (4.2%) | 2779 (100%) |
|  |  |  |  |
| Yes | 132 (86.8%) | 20 (13.2%) | 152 (100%) |
|  |  |  |  |

McNemar-test: chi^2^: 0.90 ; degrees of freedom:1 ; Prob>chi^2^: 0.343
